# Cognitive impairment in long-living adults: a genome-wide association study, polygenic risk score model and molecular modeling of the APOE protein

**DOI:** 10.3389/fnagi.2023.1273825

**Published:** 2023-10-26

**Authors:** D. A. Kashtanova, A. A. Mamchur, I. H. Dzhumaniyazova, M. V. Ivanov, V. V. Erema, E. A. Zelenova, A. Y. Yakovchik, M. S. Gusakova, A. M. Rumyantseva, M. V. Terekhov, L. R. Matkava, A. A. Akopyan, I. D. Strazhesko, V. S. Yudin, V. V. Makarov, S. A. Kraevoy, O. N. Tkacheva, S. M. Yudin

**Affiliations:** ^1^Federal State Budgetary Institution “Centre for Strategic Planning and Management of Biomedical Health Risks”, Federal Medical Biological Agency, Moscow, Russia; ^2^Russian Clinical Research Center for Gerontology, Pirogov Russian National Research Medical University of the Ministry of Healthcare of the Russian Federation, Moscow, Russia

**Keywords:** cognitive impairment, MMSE, long-living individuals, GWAS, polygenic risk score, APOE, dementia, molecular dynamics

## Abstract

**Background:**

Cognitive impairment is an irreversible, aging-associated condition that robs people of their independence. The purpose of this study was to investigate possible causes of this condition and propose preventive options.

**Methods:**

We assessed cognitive status in long-living adults aged 90+ (*n* = 2,559) and performed a genome wide association study using two sets of variables: Mini-Mental State Examination scores as a continuous variable (linear regression) and cognitive status as a binary variable (> 24, no cognitive impairment; <10, impairment) (logistic regression).

**Results:**

Both variations yielded the same polymorphisms, including a well-known marker of dementia, rs429358in the APOE gene. Molecular dynamics simulations showed that this polymorphism leads to changes in the structure of alpha helices and the mobility of the lipid-binding domain in the APOE protein.

**Conclusion:**

These changes, along with higher LDL and total cholesterol levels, could be the mechanism underlying the development of cognitive impairment in older adults. However, this polymorphism is not the only determining factor in cognitive impairment. The polygenic risk score model included 45 polymorphisms (ROC AUC 69%), further confirming the multifactorial nature of this condition. Our findings, particularly the results of PRS modeling, could contribute to the development of early detection strategies for predisposition to cognitive impairment in older adults.

## Graphical abstract

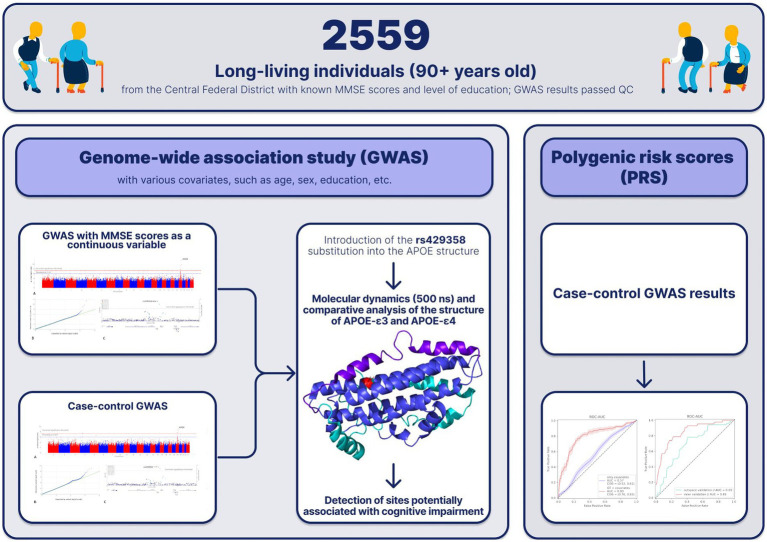

## 1. Introduction

Globally, the number of long-living adults (aged 90 years and older) has been increasing dramatically. These individuals exhibit a degree of genetic homogeneity and rarely carry pathogenic gene variants associated with the early onset of life-threatening diseases, including cognitive impairment (CI) (Balabanski et al., 2020). Therefore, examining their genetic makeup could provide valuable insights into the underlying mechanisms of CI. Several studies have focused on social factors, such as education, as well as the clinical causes and manifestations of CI in long-living adults. However, there are still significant gaps in our understanding of the genetic mechanisms underlying this condition. Han et al. (2020) performed a genome-wide association study in a Chinese cohort of long-living individuals and found that the following variants were protective against CI: rs13198061 in ESR1; rs56368572 in CTNND2; rs954303 near RNU4-58P; and rs939432 in RYR3. Based on polygenic risk scores (PRSs), the authors concluded that ESR1 and RYR3 play an important role in CI pathogenesis. These results are based, in part, on genotype imputation. Nonetheless, they add to our understanding of the genetics of CI.

Variations in the apolipoprotein E-encoding APOE gene on chromosome 19 have been recurring findings in studies on the phenomena of longevity and healthy cognitive functioning (Huang and Mahley, 2014). Disrupted catabolism and transport of lipids underlie many aging-associated neurodegenerative and cardiovascular diseases (Sato and Morishita, 2015). APOE, TOMM40, and APOC1 in its vicinity have also been associated with successful aging and neurodegenerative disorders (Zhou et al., 2014; Huang et al., 2016). Humans carry three APOE alleles: ε2, ε3, and ε4 (mean frequency = 6.4%, 78.3%, and 14.5%, respectively) (Eisenberg et al., 2010). Most studies on the genetics of long-living individuals have detected SNPs s429358 and rs7412 (Deelen et al., 2019). The ε4 allele originates from rs429358, which lowers the chances of living to 90+ years. The ε2 allele originates from rs7412, which increases the chances of living to 90+ years. These SNPs have also been associated with a genetic predisposition to Alzheimer’s disease (Bertram et al., 2007).

АРОЕ is a 34 kDa globular protein (Chou et al., 2005) with three structural domains: the N-terminal domain, the C-terminal domain, and the hinge domain (Chen et al., 2011). Due to its atypical structure, there is only one full-length experimental model of this protein available from the Protein Data Bank—2L7B (Chen et al., 2011). In the absence of a clearly determined structure of the full-length protein structure, the molecular modeling techniques used in this study seem optimal for analyzing the molecular mechanisms underlying the functional changes induced by the rs429358 substitution.

Currently, testing for the genetic susceptibility to cognitive disorders, particularly Alzheimer’s disease, in older adults relies on early detection of the APOE ε4 allele. However, as mentioned above, cognitive impairment is associated with several other genes, including TOMM40, APOC1, ESR1, RYR3, etc. In this study, we used a multifactorial approach to cognitive impairment, which allowed us to better understand the predisposition to cognitive disorders.

In our previous study of long-living adults, we reported associations between cognitive impairment and several factors, such as social (age, income, social involvement, etc.) and physiological (family history; gynecologic history, including age at onset of menopause; physical activity, etc.) (Kashtanova et al., 2022). However, the genetic traits of Russian long-living adults and the association of these traits with cognitive impairment have not been extensively studied. It is crucial to identify the underlying mechanisms of cognitive impairment in people of different ages and to determine the most appropriate individual prevention measures for healthy cognitive functioning and successful longevity. This study aimed to bridge this gap and provide comprehensive data on the molecular and genetic mechanisms underlying cognitive impairment in Russian long-living adults.

## 2. Methods

### 2.1. Participants, assessment methods, and medical histories

For a comprehensive description of the study design, see the previous article (Kashtanova et al., 2022). Participants in this single-center, non-interventional, cross-sectional study were randomly recruited in 2019–2021 in collaboration with social services, retirement homes, geriatric centers, and other geriatric institutions in Moscow and the Moscow region. The study was open to all people aged 90 years and older who provided informed consent, except for people with mental or psychiatric disabilities. A total of 2,559 participants provided their medical history, completed geriatric scales and questionnaires, and had their biomaterials (whole blood) sampled. All procedures were performed or assisted by a trained physician and a certified nurse during multiple visits to the participants’ places of residence.

The Mini-Mental State Examination (MMSE) was used to assess cognition: ≤ 9 points indicated cognitive impairment; > 24 points, no cognitive impairment (Folstein et al., 1975; Upton, 2013). A binary approach with two opposing variables (cognitive impairment/no cognitive impairment) was applied to avoid cofactor effects (such as, sensory deficits, increased fatigability, etc.) and to manage hard-to-interpret cutoff values.

After the initial assessment (365 ± 30 days), the participants or their relatives were contacted to inquire about possible adverse events, including acute conditions, hospitalization, or death. When the participants or their relatives could not be reached, social and medical services were contacted.

Preliminary analysis and data visualization were performed using Python libraries (v. 3.9.12).

### 2.2. DNA extraction, genome-wide sequencing, and quality control

The QIAamp DNA Mini Kit (Qiagen, Germany) was used for DNA extraction from the whole blood samples. The Nextera DNA Flex kit (Illumina, United States) was used to create a WGS library. The samples were sequenced to 150 bp reads and at least 30× mean depth of coverage. The Illumina Dragen Bio-IT platform (Illumina, United States) was used to align reads to the reference genome (GRCh38). Strelka2 (quality filtering) (Kim et al., 2018) was used for small-variant calling. Before individual quality control steps, all datasets were filtered using an upper threshold for missing data of 5%. Low-quality data were removed, such as those with an individual call rate <0.98; heterozygosity outliers (*F* ± 0.20); phenotype/genotype gender mismatches (females: *F* > 0.2, males: *F* < 0.2); and samples with cryptic relatedness or duplicates (PI_HAT >0.2). Variants violating the Hardy–Weinberg equilibrium (*p* < 10^−6^), variants with a call rate >0.98, multiallelic variants, and variants with a minor allele frequency <1% were also removed.

A separate group of participants was examined for the genetic variants rs3851179, rs3747742, and rs1990621, previously described as protective (Benitez et al., 2014; Santos-Rebouças et al., 2017; Li et al., 2020; Seto et al., 2021).

### 2.3. Population structure analysis

To account for population structure, a principal component analysis (PCA) was performed on a dataset of 15,000 SNPs from the Human Core Exome SNP Array (Illumina) with a frequency of less than 1% using Scikit-learn, a free machine learning library for the Python programming language. The stability of the results was confirmed in over 50 simulations (variance <5%). The first 10 principal components were used as covariates in the genome-wide association studies.

### 2.4. Genome-wide association study

Logistic and linear regressions were used in the genome-wide association study.

In the logistic regression analysis, cognitive status was encoded as two opposing values: “cognitive impairment” and “no cognitive impairment.” The following equation was used:


$$\log\left(\frac{p}{1-p}\right)=\beta_{0}+\beta_{c}*C+\beta_{g}*G$$

where: *β*_0_ = constant, *β*_c_ = coefficient of the covariate vector, *C* = covariate vector, *β*_g_ = vector of the coefficient of the genotype vector, *G* = genotype vector.

To avoid overfitting, data from 90% of the participants were randomly selected and used as a training set, while the remaining 10% were used as an additional validation set.

The following equation was used in the linear regression analysis of the MMSE score as a continuous variable:


$$Y=\beta_{0}+\beta_{c}*C+\beta_{g}*G$$

where: *β*_0_ = constant, *β*_c_ = coefficient of the covariate vector, *C* = covariate vector, *β*_g_ = vector of the coefficient of the genotype vector, *G* = genotype vector.

Non-informative SNPs were filtered out, and variant calling was optimized. The Python library (statsmodels v0.12.2) and Spark Cluster parallel processing were used for the calculation. Age, sex, and the first 10 principal components were used as covariates.

Variants were considered significant if they reached a Bonferroni threshold of *p* < 5.0 × 10^−8^. The LocusZoom JavaScript library was used to visualize regional associations.

### 2.5. Polygenic risk score

The polymorphisms identified in the genome-wide association study of the binary datasets were used to build a polygenic risk score model using the following equation:


$$\log\left(\frac{p}{1-p}\right)=\beta_{0}+\beta_{c}*C+\beta_{g}*G$$

where: *p* = probability of dementia; *β*_0_ = model constant; *β*_с_ = coefficient generated with selected covariates; *β*_g_ = coefficient generated with selected genotypes; *C* = covariate vector; *G* = genotype vector.

To factor in the population structure, age, sex, and the first 10 principal components were used as covariates.

The model was built iteratively, with more polymorphisms added in each iteration to identify the overfitting threshold resulting in a loss of accuracy on the validation dataset. The coefficients were calculated using ridge regression.

To train the logistic regression model, the sample was randomly divided into training and test sets (80% and 20%, respectively). In each iteration, the accuracy was evaluated by 10-fold cross validation on the validation dataset (20% of the training dataset) and measured by ROC AUC.

### 2.6. Polygenic risk score model validation

The PRS model was validated using additional data from 100 participants with known phenotype: 50 participants with cognitive impairment and 50 participants with no cognitive impairment, which were not used for testing or training. The above protocol was followed. The predicted polygenic risk scores were compared with the known phenotype, and the ROC AUC was used to measure the accuracy of the final PRS.

### 2.7. Molecular modeling of APOE

The NMR structure of the wild-type APOE (ε3) was obtained from the Protein Data Bank (2l7b) (Chen et al., 2011). The PyMOL (Schrödinger) mutagenesis tool was used to generate the ε4 structure. The GROMACS package (version 2020.1) (Abraham et al., 2015) and the CHARMM27 all-atom force field (Klauda et al., 2010) were used for molecular dynamics (MD) simulations. An integration time step of 2 fs was set, and 3D periodic boundary conditions were implemented. A temperature of 300 K and a pressure of 1 atm were maintained in the system through velocity rescaling (Bussi et al., 2007) and the Parrinello-Rahman algorithm (Parrinello and Rahman, 1998), respectively. The proteins were solvated [water model TIP4P (Jorgensen et al., 1998)]. A 12 Å cutoff radius was defined for the Coulombic and van der Waals interactions. Particle-mesh Ewald summation (Essmann et al., 1998) was used to compute the electrostatic interactions. Na^+^ ions were added to neutralize the system. Prior to the MD simulations, the conjugate gradient algorithm was used to minimize the energy (in 10,000 steps), followed by heating from 5 to 300 K over a period of 5 ns. For each model iteration, 500 ns MD trajectories were calculated, which totaled only 1 μs for the APOE dynamics. The MDAnalysis Python package (Michaud-Agrawal et al., 2011) and PyMOL were used for data analysis and visualization.

## 3. Results

### 3.1. The study cohort

**Figure 1:**
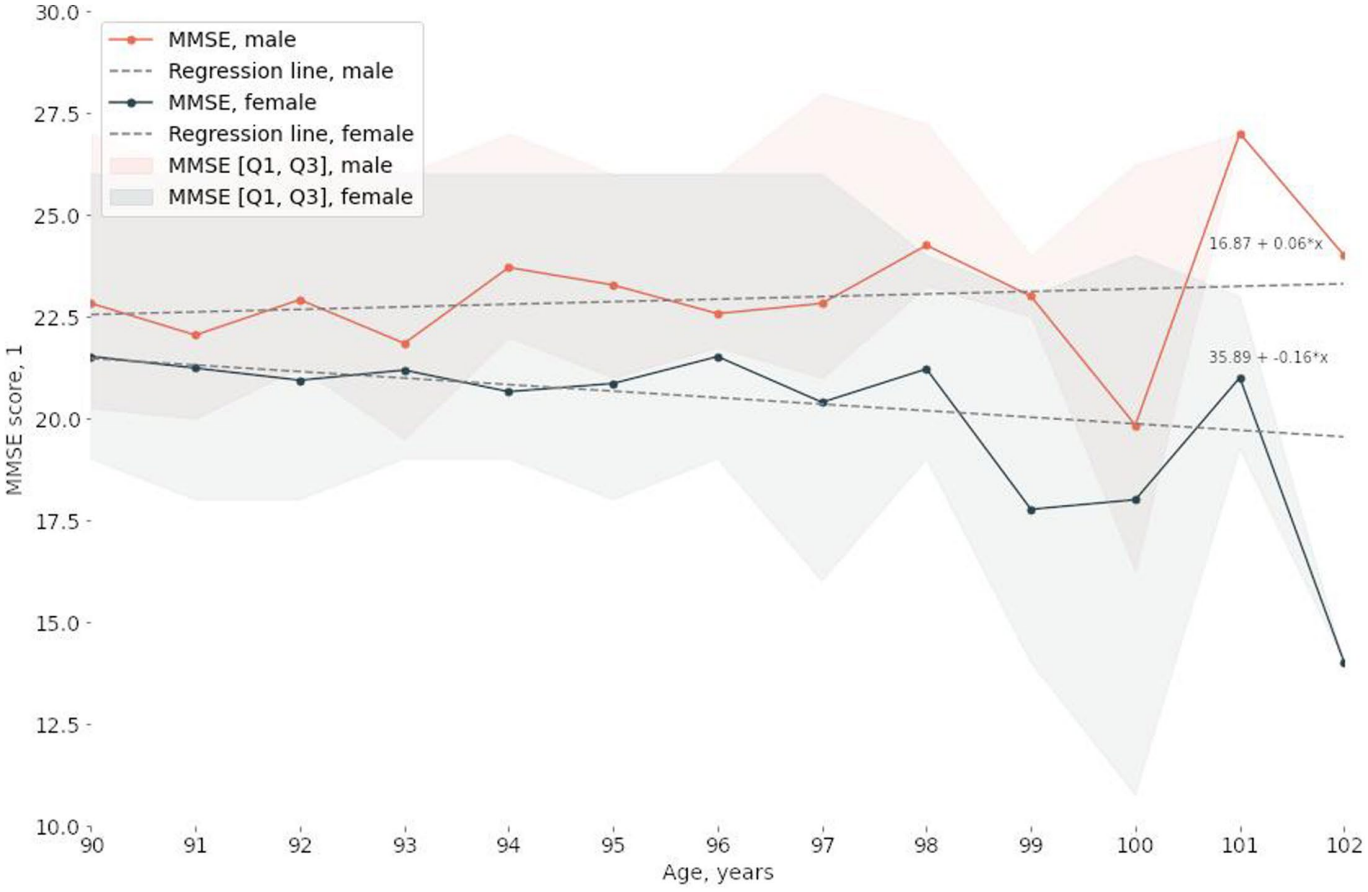
Correlations between the median MMSE score and age and sex.

The study involved 2,559 participants between the ages of 90 and 102, 75% of whom were women. The median MMSE score Was 23.0 points [19.0, 26.0]. Table 1; Supplementary Figure S1 detail the characteristics of the study participants. Sex and age significantly correlated with MMSE scores (Figure 1; Table 1; Supplementary Figure S1). The results of the statistical analysis were adjusted accordingly.

**Table 1:** Characteristics of participants from linear regression analysis of MMSE scores as a continuous variable.

Characteristic		*N*	*n*, % or median [*Q*1; *Q*3] median [*Q*1, *Q*3]	CC	*p*-value
Sex	Female	1940	23.00 [18.00; 26.00]	−1.64	1.74 × 10^−07^
	Male	619	24.00 [21.00; 27.00]	1.64	1.74 × 10^−07^
Age, years		2,559	92.00 [91.00; 94.00]	−0.10	9.0 × 10^−02^
BMI*, kg/m^2^		2,419	25.50 [23.10; 28.40]	0.21	9.1 × 10^−12^
Education*	Basic	199	199 (7.9%)	−4.24	4.06 × 10^−19^
	Secondary basic	328	328 (13.1%)	−0.28	4.65 × 10^−01^
	Secondary complete	424	424 (16.9%)	−1.30	1.55 × 10^−04^
	Secondary vocational	170	170 (6.8%)	0.21	6.79 × 10^−01^
	Advanced vocational	434	434 (17.3%)	−0.03	9.25 × 10^−01^
	Undergraduate	31	31 (1.2%)	0.42	7.21 × 10^−01^
	Graduate	857	857 (34.1%)	1.99	2.82 × 10^−13^
	Post-graduate	67	67 (2.7%)	2.63	1.05 × 10^−03^
Depression* (GDS-5; >2 points)		2,503	855 (34.2%)	−0.88	1.2 × 10^−24^
Dependence ADL*	Independent (Barthel score of >95)	215	215 (8.6%)	3.77	1.13 × 10^−14^
	Dependent (Barthel score of ≤95)	2,298	2,298 (91.4%)	−3.77	1.13 × 10^−14^
Diabetes mellitus (type 2)*		2,559	378 (14.8%)	−0.4581	0.228
Hypertension*		2,559	2,274 (88.9%)	1.1648	0.008
Dyslipidemia*	Total cholesterol ≥5.2 mmol/L	2,533	914 (36.1%)	0.9349	0.001
Total cholesterol*, mmol/L		2,533	4.77 [3.95; 5.57]	0.31	4.5 × 10^−03^
HDL*, mmol/L		2,532	1.25 [1.03; 1.52]	2.72	2.4 × 10^−13^
LDL*, mmol/L		2,524	2.86 [2.21; 3.56]	0.02	9.0 × 10^−01^
Lp(a)*, mg/dL		2,543	128.0 [111.0; 147.0]	0.05	3.22 × 10^−25^
Triglyceride*, mmol/L		2,507	1.18 [0.9; 1.54]	0.6	0.008

*Coefficients and *p*-values of age and sex were not adjusted; coefficients and *p*-values of other characteristics (*) were adjusted for age and sex. ADL, activity of daily-living; BMI, body mass index; СС, correlation coefficient; GDS-5, geriatric depression scale-5; MMSE, Mini-Mental State Examination; *n*, the number of participants with the characteristic under consideration; *N*, the number of participants with the known value for the characteristic under consideration; LDL, low-density lipoproteins; HDL, high-density lipoproteins; Lp(a), lipoprotein (a).

### 3.2. GWAS: a linear regression model based on MMSE scores

**Figure 2:**
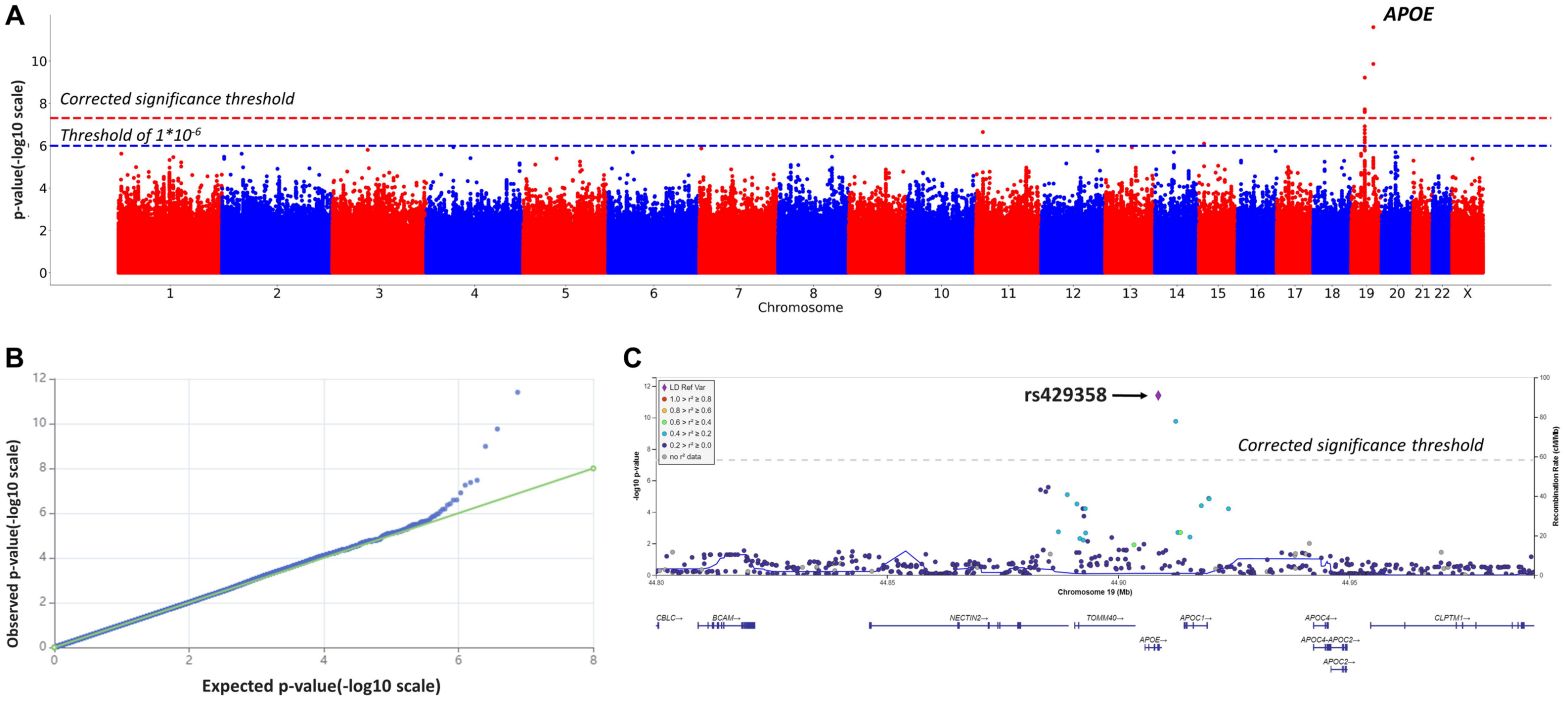
Manhattan plot **(A)**, QQ plot **(B)**, and regional association plot **(C)** for the linear regression model based on MMSE scores as a continuous variable (adjusted for age, sex, and the first 10 principal components). **(A)** Manhattan plot of −log10 *p*-values of common variants. The dashed red line represents a Bonferroni threshold of (−log10(5 × 10^−8^)). The dashed blue line represents a threshold of (−log10(1 × 10^−6^)). **(B)** GWAS QQ-plot. Most of the observed and expected *p*-values were identical, indicating the validity of the GWA model. **(C)** Regional association plot for the locus on chromosome 19 (chromosome 19:44,878,048–44,944,779) that contains all significant SNPs. The color indicates the strength of linkage disequilibrium between the lead SNP, rs429358, and other SNPs in this region. The dashed line represents a Bonferroni threshold of (−log10(5 × 10^−8^)).

After quality filtration, 8,455,468 variants were tested. Eight variants located on chromosomes 10 and 19 reached the genome-wide significance threshold. Table 2; Figure 2 present the results of the linear regression modeling.

**Table 2:** Summary table of GWAS results.

Chr	Position	AF	LR coefficient	*p*-value	Statistics in the Hardy–Weinberg test	Hardy–Weinberg test (*p*-value)	Gene	Gene variant	SNP
**GWAS, MMSE score as a continuous variable**									
chr19	44,908,684	0.077	−2.49	2.28 × 10^−12^	2.88	0.24	APOE	Missense variant	rs429358
chr19	27,353,865	0.014	−4.45	2.08 × 10^−8^	0.55	0.76		Intergenic variant	rs145461979
chr19	44,912,456	0.063	−2.49	1.29 × 10^−10^	1.03	0.60	APOE	Downstream gene variant	rs10414043
chr19	27,359,319	0.014	−4.42	2.35 × 10^−8^	0.55	0.76		Intergenic variant	rs78741720
chr19	44,906,745	0.061	−2.49	2.43 × 10^−10^	1.51	0.47	APOE	Intron variant	rs769449
chr19	27,355,589	0.026	−3.35	1.73 × 10^−8^	1.88	0.39		Intergenic variant	rs113472381
chr19	27,369,272	0.018	−4.49	5.41 × 10^−10^	0.82	0.66		Intergenic variant	rs10048455
chr10	106,063,954	0.012	−5.18	4.02 × 10^−9^	0.35	0.84		Intergenic variant	rs193174984
**GWAS, “case-control”. “Case” = MMSE <10; “Control” = MMSE >24**									
chr19	44,908,684	0.067	1.24	7.71 × 10^−10^	2.26	0.32	APOE	Missense variant	rs429358
chr19	27,349,377	0.037	1.41	1.95 × 10^−8^	1.73	0.42		Intergenic variant	rs113288717
chr19	44,912,456	0.055	1.28	4.51 × 10^−9^	1.93	0.38	APOE	Downstream gene variant	rs10414043
chr19	44,906,745	0.052	1.25	1.74 × 10^−8^	1.65	0.44	APOE	Intron variant	rs769449
chr19	27,355,589	0.032	1.58	1.61 × 10^−9^	1.30	0.52		Intergenic variant	rs113472381
chr19	27,369,272	0.020	1.78	1.59 × 10^−8^	0.50	0.78		Intergenic variant	rs10048455
chr4	49,710,764	0.018	1.87	3.62 × 10^−8^	0.38	0.83		Regulatory region variant	rs1293508533
chr1	221,475,211	0.771	−0.50	3.32 × 10^−8^	1033.60	3.6 × 10^−225^		Intergenic variant	rs11118728

Chr, chromosome number; AF, allele frequency; LR coefficient, logistic regression coefficient.

SNPs on chromosome 19 were of particular interest, as seen in Figure 2C. SNPs rs145461979, rs78741720, rs113472381, and rs10048455 in intergenic regions, as well as rs193174984 on chromosome 10, had not been previously described. SNPs rs429358 (in an APOE exon; chr19:44908684, GRCh38; *p*-value = 2.2808 × 10^−12^) and rs769449 (in an APOE intron; chr19:44906745, GRCh38; *p*-value = 2.4323 × 10^−10^) are particularly relevant. Substitutions at these positions and at rs10414043 (in a non-coding region; chr19:44912456, GRCh38; *p*-value = 1.2851 × 10^−10^) were associated with lower MMSE scores. The regression coefficient for rs429358 was −2.4882; for rs769449, −2.4867; and for rs10414043, −2.4925. Linkage disequilibrium (LD) between rs429358 and significant SNPs in its vicinity did not exceed 0.4 (*R*^2^).

Given reported associations between education and healthy cognition, GWAS results were adjusted for education (Supplementary material, education-adjusted GWAS results, Supplementary Table S2; Supplementary Figures S2, S3). The adjusted and non-adjusted GWAS results were identical (Table 2; Figure 2, non-adjusted GWAS results; Supplementary Table S2; Supplementary Figure S3, adjusted GWAS results). Therefore, no adjustment for education was further applied (significant APOE polymorphisms showed similar associations with MMSE scores).

### 3.3. Genome-wide association study using logistic regression modeling based on MMSE scores

**Figure 3:**
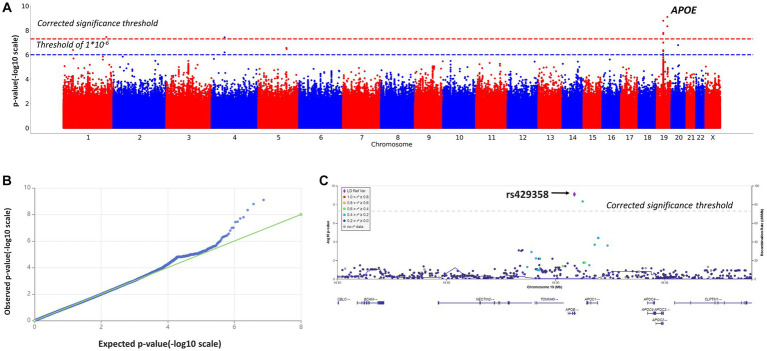
Manhattan plot **(A)**, QQ plot **(B)**, and regional association plot **(C)** for the logistic regression model based on MMSE scores as a binary variable (adjusted for age, sex, and the first 10 principal components). **(A)** Manhattan plot of −log10 *p*-values of common variants. The dashed red line represents a Bonferroni threshold of (−log10(5 × 10^−8^)). The dashed blue line represents a threshold of (−log10(1 × 10^−6^)). **(B)** GWAS QQ-plot. Most of the observed and expected *p*-values were identical, indicating the validity of the GWA model. **(C)** Regional association plot for the locus on chromosome 19 (chromosome 19:44,878,048–44,944,779) that contains all significant SNPs. The color indicates the strength of linkage disequilibrium between the lead SNP, rs429358, and other SNPs in this region. The dashed line represents a Bonferroni threshold of (−log10(5 × 10−8)).

In addition, we performed a genome-wide association study of those participants, whose cognitive assessments fell into two opposite categories: cognitive impairment (MMSE <10) and no cognitive impairment (MMSE >24). The characteristics of this sub-cohort (*n* = 1,155) are described in Supplementary Table S1. The results are provided in Table 2; Figure 3. A total of 9,287,600 polymorphisms were analyzed. After quality filtration, 8,505,513 of them were tested in a binary logistic regression model. Eight polymorphisms across three chromosomes (1, 4, and 19) reached the genome-wide significance threshold.

These results were largely identical to the results of the genome-wide association study based on MMSE scores as a continuous variable (Table 2). The logistic regression model revealed significant associations between cognitive impairment (MMSE <10 points) and rs10414043 (*p*-value = 4.5*10^−9^; coeff. =1.2811), rs429358 (*p*-value = 7.7*10^−10^; coeff. = 1.2419), and rs769449 (*p*-value = 1.7 × 10^−8^, coeff. = 1.2503). These SNPs were generated in both linear (continuous variable) and binary models; hence, they were further studied in more detail.

Additionally, we detected CI-associated SNPs with genome-wide significance, rs11118728 (chromosome 1), rs1293508533 (chromosome 4), and rs10048455, rs113472381, and rs113288717 (chromosome 19) (Table 2), which had not been previously described or annotated.

### 3.4. Molecular modeling of APOE

**Figure 4:**
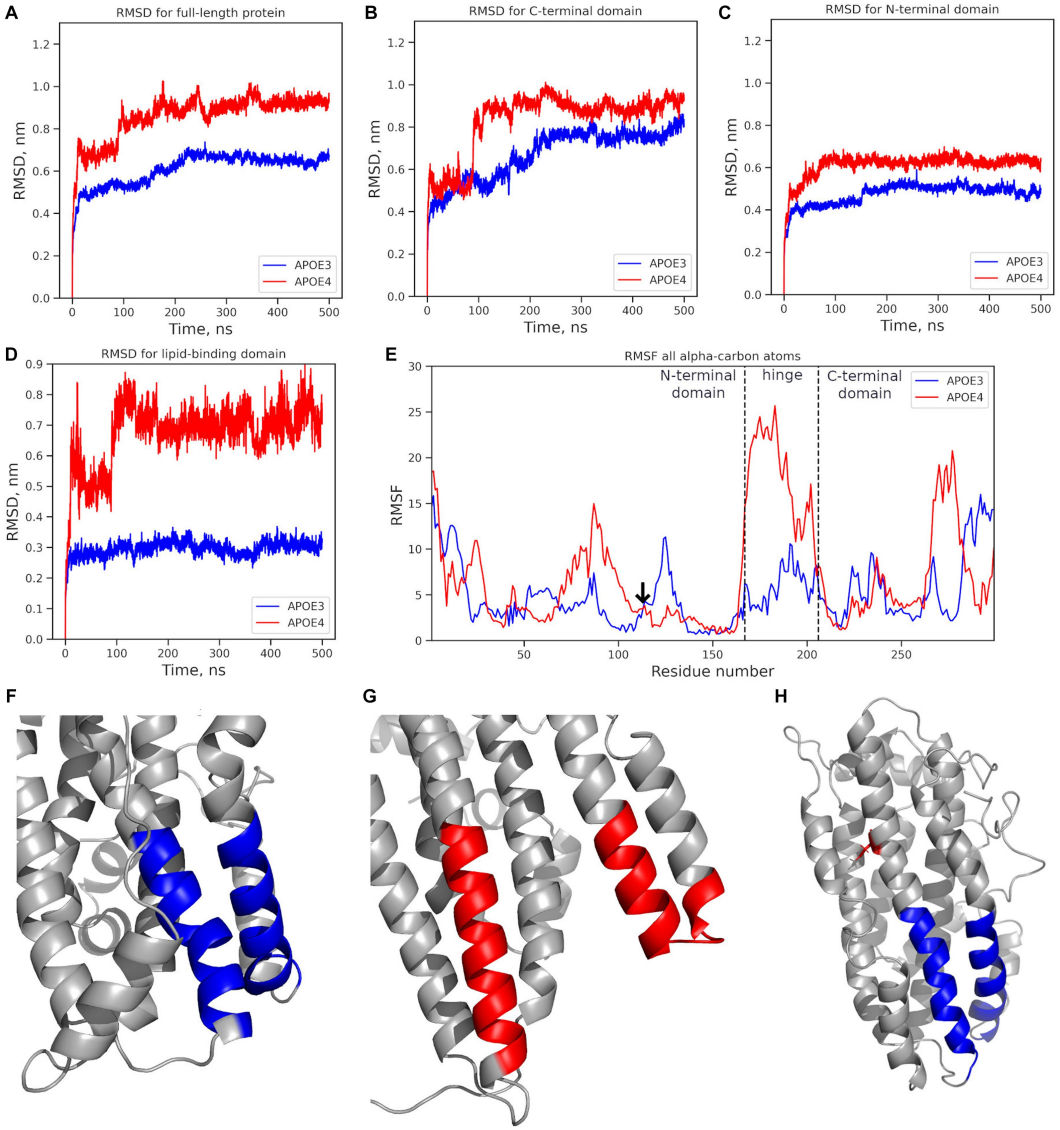
Mobility analysis of APOE isoforms: ε3 (APOE3, dark blue) and ε4 (APOE4, red). Changes in the RMSD value: **(A)** full-length protein; **(B)** the N-terminal domain; **(C)** the C-terminal domain; **(D)** the lipid-binding domain. **(E)** the RMSF for all alpha-carbon atoms. The vertical dotted line marks the edges of the N-terminal, hinge, and C-terminal domains. The arrow marks the mutation site. **(F)** Structure of the ε3 isoform’s lipid-binding domain in the final MD simulation frame. **(G)** Structure of the ε4 isoform’s lipid-binding domain in the final frame. **(H)** The initial protein structure; blue: the lipid-binding domain; red: the location of the C112R substitution introduced to from the APOE4 isoform. ^*^APOE3, APOE ε3 isoform; APOE4, APOE ε4 isoform.

The exon-located rs429358 leads to the C112R substitution. To understand the molecular mechanisms underlying its effects, we compared the ε3 (wild-type) and ε4 (rs429358) protein isoforms. The tertiary structure of the protein and the site of C112R introduction are shown in Supplementary Figure S4. Further relaxation of the protein and the introduction of the C112R substitution in 2L7B (from the Protein Data Bank) had no discernible effect on the structure of APOE. However, a gain of a single positive charge in ε4 led to both changes in the net charge of the protein (−5 in ε3; −4, in ε4) and changes in the electrostatic interaction map, affecting the interactions between the APOE domains. Molecular dynamics (MD) simulations were used to analyze the behavior of all APOE isoforms in a solution, and the root-mean-square deviation (RMSD) was calculated. In the 500 ns MD simulations, the APOE isoforms demonstrated different degrees of mobility (Figure 4). Notably, ε3 remained the most stable throughout the simulation process (APOE3, Figure 4A), whereas ε4 showed the greatest deviation from its original structure (APOE4, Figure 4A).

The mobility of individual domains was also analyzed (Figures 4B,C), including the root mean square fluctuation (RMSF) of the amino acids (Figure 4D). The C-terminal domain had the highest RMSD value (0.8–1.1 nm) and contributed most to the deviation from the original protein structure (Figure 4B). The RMSD value of the N-terminal domain did not exceed 0.7 nm (Figure 4C). In APOE2 and APOE3, the N-terminal domains showed almost similar changes in the RMSD values. In APOE4, the RMSD value of the N-terminal domain was 0.15 nm higher. However, fluctuation analysis did not reveal any significant differences in the mobility of the N-terminal domains (Figure 4E). RMSF analysis revealed significantly more mobile hinge and C-terminal domains in APOE4 compared to “wild-type” APOE3 (Figure 4B), with maximum mobility at amino acids 260–280 (Figure 4E).

The structure of the lipid-binding domain described by Frieden et al. (2017) at positions 88–104 and 251–266 was examined in detail. The lipid-binding domain in the ε4 isoform showed structural changes in the MD simulations, while in the ε3 isoform, it remained very stable (Figure 4D). The most dramatic changes were observed at amino acids 251–266 in the highly mobile C-terminal domain. The RMSF analysis of the amino acids 251–266, where the helical unwinding occurred, did not provide definitive results (Figure 4E). However, the region 260–266 was significantly more mobile in APOE4 than in other isoforms.

No such changes were observed in the region, associated with LDL-binding (amino acids 140–150) (Frieden et al., 2017) or β-amyloid-binding (amino acids 133–135 and 150–161) (Tsiolaki et al., 2019) in any APOE isoform.

### 3.5. Associations between APOE genotypes, lipid metabolism, and 1 year mortality

The results of the molecular modeling suggest an association between the APOE isoforms and lipid metabolism, since the structural deviation of the lipid-binding domain in APOE would disrupt the main function of the protein. Therefore, we examined the effects of just a single ε4 allele on lipid metabolism. We found that carrying even a single ε4 allele was associated with increased levels of total cholesterol (coeff. = 0.169; *p*-value = 0.013) and LDL (coeff. = 0.188; *p*-value = 9.2 × 10^−4^) and a higher atherogenic index (coeff = 0.194, *p*-value = 4.2 × 10^−4^) (Supplementary Table S3).

It is worth noting that long-living adults rarely carry the ε4 allele. In our study, the allele frequency was 0.007, regardless of cognitive status. The genome-wide association study using linear regression (*n* = 2,559; adjusted for age and sex) showed that even a single ε4 allele (rs429358) contributed to cognitive impairment in the oldest-old (coeff. = −2.4882; *p*-value = 2.2808 × 10^−12^). There was no significant difference in LDL levels between the “cognitive impairment” group and the “no cognitive impairment” group, whereas the HDL levels were significantly higher in the “no cognitive impairment” group (Supplementary Table S1). A 1-unit increase in the HDL/LDL ratio was associated with lower MMSE scores (coeff. = −0.736; *p*-value = 6.4 × 10^−8^, adjusted for age and sex). These associations may represent the mechanism underlying the neurodegenerative effects of APOE.

The ε2 allele of the APOE gene originates from the polymorphism rs7412 and is typically associated with the maintenance of cognitive functions. In our study, this allele did not reach genome-wide significance (linear regression, coeff. = 0.6597; *p*-value = 0.0342). In contrast to the ε4 allele, carrying even a single ε2 was associated with higher MMSE scores and lower total cholesterol and LDL levels (Supplementary Table S3).

One-year mortality was known for 1,350 participants. Carrying even a single ε4 allele was associated with an 80% increase in mortality within 12 months of assessment (Supplementary Table S3).

The interaction between two alleles had an effect on MMSE scores (Supplementary Figure S5; Supplementary Table S4). The ε3/ε4 combination posed the highest risk of cognitive impairment (MMSE <10) (OR = 3.15; *p*-value =1.16 × 10^−7^). Analysis of MMSE scores as a continuous variable showed that the median MMSE score decreased in carriers of ε3/ε4, ε2/ε4, and ε4/ε4. However, the statistical significance of this decrease is difficult to assess, since only 9 participants were carriers of ε4/ε4.

### 3.6. The ε4/ε4 homozygous combination analysis

Nine ε4/ε4-carriers (3 men and 6 women between the ages of 90 and 95) were analyzed in more detail. Although the median MMSE score was lower in this group, only one carrier (a 91 years-old male; MMSE = 4 points) exhibited clear signs of cognitive impairment. With the exception of this individual, all other carriers had substitutions at loci rs3851179 (intergenic, chromosome 11), rs3747742 (TREML2 missense variant, chromosome 6), and rs1990621 (intergenic, chromosome 7), which previously have been shown to be protective against cognitive impairment (Seto et al., 2021). Notably, in the entire cohort, these substitutions had no significant effect on MMSE scores, further highlighting the multifactorial and polygenic nature of cognitive impairment.

### 3.7. Polygenic risk score model (PRS model)

The binary GWAS results were used to build a polygenic risk score model for a genetic predisposition to cognitive impairment in adults aged 90+ years.

**Figure 5:**
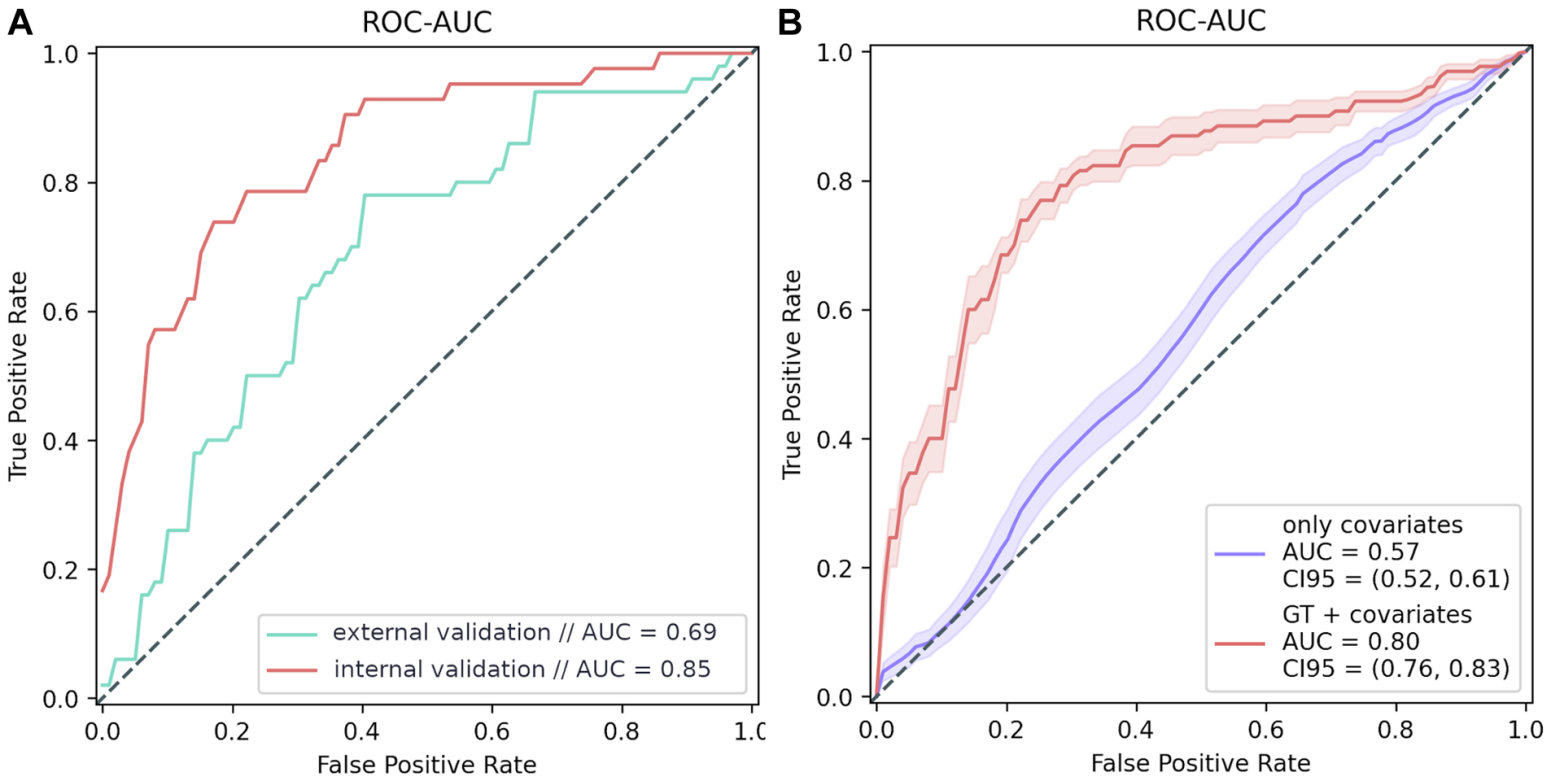
PRS modeling results. **(A)** Model testing results: internal validation: 20% of the GWAS data; external validation: additional data from 100 participants, whose data were not used for testing. **(B)** Model training results. The purple line shows the ROC curve of the covariate-based model (age and sex); the red line represents the ROC curve of the model based on both covariates and genotype.

The trained model generated a ROC AUC of 84.8% (*F*1 = 57.4%; precision = 47%; recall = 73.8%) (Figure 5). Overall, the PRS model included 45 polymorphisms (Supplementary Table S5). Notably, some of the most significant SNPs in the model did not reach the standard genome-wide significance threshold.

The final PRS model generated a ROC AUC of 69% on the external validation set which included 76 women and 24 men in age 92 (91–94) (*F*1 = 61.7%, precision = 65.9%, recall = 58%) (Figure 5A). We can conclude that the PRS model is highly specific and sensitive for assessing the risk of cognitive impairment. It is worth mentioning that models based on genetic traits are 23% more accurate risk predictors than those based on only other characteristics, such as age and sex, which are significantly associated with dementia (Figure 5B).

## 4. Discussion

Here, we present the findings from a genome-wide association study on cognitive impairment in Russian long-living adults and the results of molecular modeling of the APOE protein. We detected recurring polymorphisms on chromosome 19 in the non-coding region upstream of the APOC1 gene (rs10414043) and in the APOE gene (rs429358 and rs769449) and identified a possible mechanism whereby these substitutions contribute to cognitive impairment. SNPs associated with cognitive impairment have been well studied. However, in this study, we examined them from a different perspective, i.e., as factors contributing to cognitive impairment in long-living adults, and confirmed their significance in the Russian population.

rs10414043 G>A is located in a non-coding region on chromosome 19. The functional significance of this SNP can be speculated based on its effect on the expression of the APOC1 and TOMM40 genes, where it occupies regulatory regions. It has also been shown to be associated with changes in the volume of the hippocampus and amygdala, the most important parts of the limbic system (Yuan et al., 2019). A smaller hippocampus and amygdala (along with other parts of the brain) are predictors of Alzheimer’s disease in patients with cognitive impairment (Tabatabaei-Jafari et al., 2019).

Polymorphisms in APOE have been associated with life expectancy (Sebastiani et al., 2019) and cognitive status (Yamazaki et al., 2019). rs429358 and rs7412 are the most typical of these phenotypes.

rs429358 T>C is located at chr19: 44908684 in the 4th exon of APOE. It causes a cysteine-to-arginine substitution at amino acid 112 in APOE. This substitution can disrupt the unfolding of the protein and affect its affinity (Chen et al., 2021), leading to the formation of the ε4 isoform associated with Alzheimer’ disease. This association might be caused by the effect of the ε4 isoform, which has been shown to increase tau protein phosphorylation in a murine model (Brecht et al., 2004), and accelerate beta-amyloid deposition in the early stages of Alzheimer’s disease (Hudry et al., 2013).

rs769449 G>A (in an APOE intron) has not been described in detail and is less well known. Carriers of this polymorphism have increased levels of phosphorylated tau protein in the cerebrospinal fluid (Cruchaga et al., 2013) and blood serum (Huang et al., 2022). Increased serum levels of phosphorylated tau protein are the primary marker of Alzheimer’s disease. Moreover, a sharp decline in MMSE scores in rs769449 carriers was observed over a 100 months period (Huang et al., 2022). However, the contribution of this polymorphism to cognitive impairment remains unclear.

According to the Genome Aggregation Database (gnomAD; GnomADv2.1.1), the typical allele frequencies (IF) for ε2 (rs7412), ε3 (wild type), and ε4 (rs429358) in a mixed-age population are: 6.5%, 79.2%, and 14.3%, respectively. In our study, these alleles occurred with a frequency of 10.1%, 82.2%, and 7.6%, respectively. The ε2 allele was much more common in the study cohort than in the general population, making the rs7412 substitution a potential genetic marker for longevity. However, this variant is also known as a risk factor for cardio-vascular diseases, such as hypercholesterolemia (Garatachea et al., 2015). However, the ε4 allele was significantly less common in the cohort of long living adults than in the general population, as confirmed by published data (Garatachea et al., 2015).

### 4.1. Analysis of the APOE protein structure

Changes in the structure of the APOE protein, caused by a single-nucleotide substitution, alter its biophysical and biochemical properties, possibly accounting for its association with Alzheimer’s disease (Huang et al., 2004). However, the rs429358 substitution in the N-terminal domain, leading to the formation of the ε4 isoform, had no significant impact on the protein structure. Moreover, there were no differences in the substitution site fluctuations between the APOE isoforms (Figure 4E).

However, shifts in amino acid interactions, particularly salt bridges, caused by the gain or loss of a charged arginine, led to changes in the contacts between the APOE domains and their mobility. Our results, therefore, suggest that the ε4 isoform deviates the most from its original structure (Figure 4A), due to a simultaneous increase in the mobility of both the N- and C-terminal domains (Figures 4B,C). This isoform also showed increased mobility in the hinge domain (Figure 4E), which had been previously demonstrated in comparative studies of the APOE4 and the wild-type isoform structures (Ray et al., 2017). However, the authors also observed helix formation, and, hence, reduced conformational mobility at 270–280 in ε4, which contradicts our finding of significant fluctuations in these amino acids (Figure 4E).

We propose that increased conformational mobility of the 260–280 region in the C-terminal domain of APOE4 may play a role in the pathogenesis of cognitive impairment, in contrast to previous findings suggesting that the salt bridge R61-E255 stabilizes the C-terminal domain (Hatters et al., 2006). Increased conformational mobility makes the protein more available for proteolysis, resulting in the formation of truncated APOE4 fragments (Δ272–299) associated with amyloid aggregation (Harris et al., 2003). Stabilization of the C-terminal domain in APOE2 and APOE3 presumably preserves the full-length protein and improves its functionality.

Our findings also suggest that impaired lipid transporter function underlies the pathogenic effects of the APOE 4 isoform. Reduced lipid transport is the result of a low affinity of individual monomers resulting from alterations in the structure of the lipid-binding domain, as demonstrated in this study. However, the molecular nature of lipoprotein formation by the ε2 and ε4 isoforms should be studied in more detail *in silico*, *in vitro*, and *in vivo*.

### 4.2. Homozygote analysis

There were only 9 carriers of the homozygous ε4 allele among 2,559 participants. This finding is consistent with the published data that the frequency of this allele is generally lower in older-adults than in mixed-age populations. The homozygous ε4 allele could be inversely correlated with longevity (Garatachea et al., 2015). The subgroup of long-living ε4/ε4 carriers with relatively higher MMSE scores (except for one participant) is an intriguing case. This subgroup suggests that the rs429358 substitution, despite its demonstrated significance, is not a sufficient prerequisite for the formation of the cognitive decline phenotype or its sole determinant. An additional analysis of the functional pathways and PRS modeling confirmed this suggestion.

### 4.3. Polygenic risk score model

Cognitive status is a complex and heterogeneous phenotypic trait. Normal cognitive functioning in long-living adults aged 90+ could be accounted for by a large number of the so-called protective polymorphisms, each with limited individual significance (Seto et al., 2021). Given the heterogeneous nature of cognitive impairment and the polygenic nature of its inheritance, we built a polygenic risk score model for cognitive impairment in long-living adults.

Polygenic risk score modeling allows for a comprehensive genetic study of cognitive impairment and the identification of the contribution of each polymorphism to the formation of this complex trait. The most significant genes in our PRS model were *GRIK3*, *SV2C*, and *DKK3,* each containing a single intron polymorphism (Supplementary Table S5).

GRIK3 and SV2C regulate synaptic transmission. GRIK3 encodes a glutamate receptor and has previously been associated with mental disorders, particularly schizophrenia (Dai et al., 2014). Latimer et al. (2014) suggest that it is signaling through this receptor that underlies the improved cognitive performance in mice supplemented with vitamin D. Pathway enrichment analysis also showed that changes in the expression of this gene are associated with the development of familial Alzheimer’s disease (Antonell et al., 2013). The synaptic vesicle glycoprotein encoded by the SVC2 gene regulates the release of dopamine into the synaptic cleft. This process has been shown to be disrupted in Parkinson’s disease (Dunn et al., 2017).

*DKK3* is a member of the Dickkopf (Dkk) family, which is involved in embryonic development, including brain development. The product of this gene is considered by some authors to be a potential biomarker of Alzheimer’s disease in cerebrospinal fluid (Zenzmaier et al., 2009).

The polygenic risk score model included many polymorphisms located on chromosome 19. Some of them have been previously described, such as rs429358 and rs769449 in *APOE* and rs10414043 in APOC1. In addition, the model included rs7256200 (APOC1), previously associated with Alzheimer’s disease (Vogrinc et al., 2021), and rs1555789087 (TOMM40). APOC1, TOMM40, and APOE are involved in lipid metabolism and are well-known markers of cognitive status (Zhou et al., 2014; Vogrinc et al., 2021).

Notably, all genes involved in synaptic transmission had more “weight” than polymorphisms in APOE, suggesting that APOE is insufficient as a single genetic predictor of dementia and further highlighting the importance of a polygenic approach to risk assessment. The multifactorial character of the cognitive impairment was also confirmed by the pathway enrichment analysis presented in the Supplementary material.

## 5. Conclusion

The genome-wide association study showed that the APOE gene plays a significant role in the development of cognitive impairment in long-living adults. The molecular modeling results showed that the rs429358 polymorphism (C112R missense substitution in the APOE protein) alters protein motility and disrupts the structure of the lipid-binding domain, which can affect the affinity of APOE for lipids and reduce the efficiency of their transport. However, the presence of this substitution is not the only factor determining the phenotype of its carrier. Cognitive impairment is a multifactorial phenotype, as demonstrated by the diversity of genes included in the polygenic risk score model presented in this study. Further insight into the mechanisms and causes of the late-onset cognitive impairment observed in long-lived adults, as well as the identification of protective factors, will allow us to propose methods for early detection of dementia or even options for its treatment.

## Data availability statement

The datasets presented in this article are not readily available due to ethical restrictions to protect participant privacy. Requests to access the datasets should be directed to the corresponding author.

## Ethics statement

The studies involving humans were approved by The Local Ethics Committee of the Russian Gerontological Research and Clinical Center (Protocol No. 30, December 24, 2019). The studies were conducted in accordance with the local legislation and institutional requirements. The participants provided their written informed consent to participate in this study.

## Author contributions

DK: Conceptualization, Investigation, Methodology, Supervision, Validation, Writing – original draft, Writing – review & editing. AM: Conceptualization, Formal analysis, Methodology, Software, Validation, Visualization, Writing – original draft, Writing – review & editing. ID: Conceptualization, Formal analysis, Methodology, Software, Validation, Visualization, Writing – original draft, Writing – review & editing. MI: Data curation, Formal analysis, Software, Validation, Visualization, Writing – original draft, Writing – review & editing. VE: Conceptualization, Methodology, Validation, Visualization, Writing – original draft, Writing – review & editing. EZ: Formal analysis, Software, Validation, Writing – original draft, Writing – review & editing. AY: Validation, Writing – original draft, Writing – review & editing. MG: Conceptualization, Methodology, Validation, Writing – original draft, Writing – review & editing. AR: Data curation, Investigation, Writing – original draft. MT: Visualization, Writing – original draft. LM: Writing – original draft, Writing – review & editing. AA: Conceptualization, Investigation, Resources, Writing – review & editing. IS: Conceptualization, Investigation, Resources, Writing – review & editing. VY: Methodology, Project administration, Supervision, Writing – review & editing. VM: Project administration, Writing – review & editing. SK: Project administration, Supervision, Writing – review & editing. OT: Project administration, Supervision, Writing – review & editing. SY: Project administration, Supervision, Writing – review & editing.

## Abbreviations

MMSE: Mini-Mental State Examination
APOE3: Apolipoprotein E, isoform ε3
APOE4: Apolipoprotein E, isoform ε4
SNP: Single nucleotide polymorphism
NTD: N-terminal domain
CTD: C-terminal domain
