# Supplementary material for: Cognitive impairment in long-living adults: a genome-wide association study, polygenic risk score model and molecular modeling of the APOE protein
Source: Front Aging Neurosci. 2023 Oct 26;15:1273825. doi: 10.3389/fnagi.2023.1273825 (PMC10637623; doi:10.3389/fnagi.2023.1273825)
Supplement: Supplementary file 1 [file Data_Sheet_1.docx]

**Supplement**

**Characteristics of the participants**


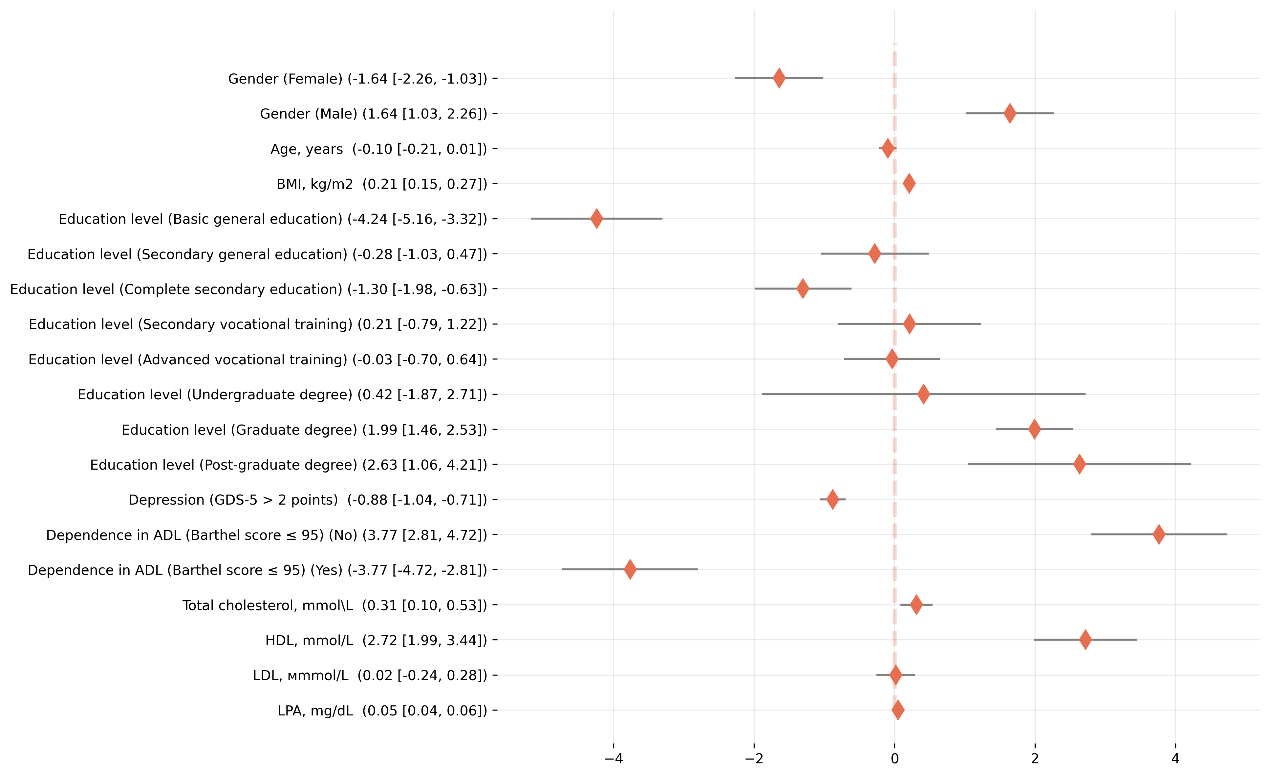


**Figure** S1. Visualization of significant factors contributing to cognitive impairment in long-living adults.

**GWAS with a binary variable**

**Table S1.** Characteristics of participants from logistic regression analysis (with cognitive status as a binary variable)

| **Characteristic** | | **N** | **n% or Median [Q1, Q3] in CI group (MMSE<10)** | **n% or Median [Q1, Q3] in no CI group (MMSE>24)** | **OR** | **p-value** |
| --- | --- | --- | --- | --- | --- | --- |
| **Sex** | Female | 859 | 153 (17.8%) | 706 (82.2%) | 2.27 | 2.91*10^-04^ |
|  | Male | 296 | 26 (8.8%) | 270 (91.2%) | 0.44 | 2.91*10^-04^ |
| **Age, years** |  | 1155 | 92.00 [91.00, 94.00] | 92.00 [90.00, 94.00] | 1.05 | 1.1*10^-01^ |
| **BMI*, kg/m^2^** |  | 1098 | 23.70 [20.95, 27.30] | 25.80 [23.60, 28.90] | 0.88 | 9.0*10^-09^ |
| **Education*** | Basic | 61 | 25 (41.0%) | 36 (59.0%) | 5.26 | 4.68*10^-09^ |
|  | Secondary basic | 110 | 13 (11.8%) | 97 (88.2%) | 0.84 | 5.78*10^-01^ |
|  | Secondary complete | 157 | 34 (21.7%) | 123 (78.3%) | 1.85 | 4.74*10^-03^ |
|  | Secondary vocational | 65 | 7 (10.8%) | 58 (89.2%) | 0.75 | 4.93*10^-01^ |
|  | Advanced vocational | 189 | 27 (14.3%) | 162 (85.7%) | 1.06 | 7.94*10^-01^ |
|  | Undergraduate | 17 | 3 (17.6%) | 14 (82.4%) | 1.14 | 8.41*10^-01^ |
|  | Graduate | 465 | 37 (8.0%) | 428 (92.0%) | 0.44 | 4.52*10^-05^ |
|  | Post-graduate | 52 | 3 (5.8%) | 49 (94.2%) | 0.45 | 1.89*10^-01^ |
| **Depression***  **(GDS-5; >2 points)** |  | 368 | 93 (25.3%) | 275 (74.7%) | 1.37 | 2.0*10^-08^ |
| **Dependence ADL*** | Dependent (Barthel score of ≤ 95) | 984 | 175 (17.8%) | 809 (82.2%) | 12.50 | 3.49*10^-04^ |
| **Total cholesterol*, mmol/L** |  | 1143 | 4.75 [4.07, 5.38] | 4.87 [3.97, 5.71] | 0.89 | 1.0*10^-01^ |
| **HDL*, mmol/L** |  | 1142 | 1.18 [0.97, 1.44] | 1.31 [1.07, 1.60] | 0.26 | 1.1*10^-07^ |
| **LDL*, mmol/L** |  | 1139 | 2.98 [2.42, 3.56] | 2.89 [2.20, 3.61] | 1.05 | 5.2*10^-01^ |
| **Lp (a)*, mg/dl** |  | 1149 | 115.00 [101.00, 133.75] | 134.00 [115.00, 154.00] | 0.97 | 2.2*10^-16^ |

*Note* Coefficients and p-values of age and sex were not adjusted; coefficients and p-values of other characteristics (*) were adjusted for age and sex. ADL: Activity of Daily-Living; BMI: Body Mass Index; СС: Correlation Coefficient; GDS-5: Geriatric Depression Scale – 5; MMSE: Mini-Mental State Examination; n; the number of participants with then characteristic under consideration, N: the number of participants with the known value for the characteristic under consideration; LDL: low-density lipoproteins; HDL: high-density lipoproteins; Lp(a): Lipoprotein (a).*

**GWAS results adjusted for education**

The results were adjusted for the level of education as a known factor of CI, where: 0=basic general education; 1=secondary general education, secondary education + vocational; 2=complete secondary education; 3= complete secondary education + advanced vocational training; 5=undergraduate degree (3-4 years at a university/college); 6=graduate degree; and 7=postgraduate degree. The adjustment was applied in the following manner: 5 education points = +0 points to the overall MMSE score; 2–5 education points= +1 to the overall MMSE score; 0–1 education points= +2 to the overall MMSE score. The MMSE score remained unchanged if education information was not provided. The adjusted MMSE score could not exceed 30 points. This approach was developed and successfully applied by Belsa et al. ([Clinical validity of the “mini-mental state” for Spanish speaking communities. Neuropsychologia. 2001;39: 1150–1157.](http://paperpile.com/b/SBIOd0/QCiS)).

Education was correlated with the median MMSE score in 2510 participants (Figure S2).


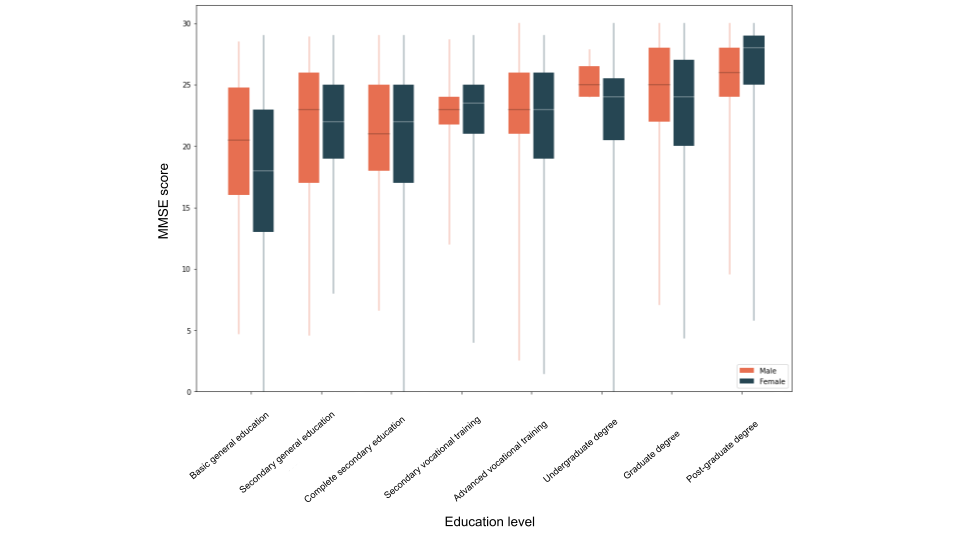


**Figure S2.** Correlation between the MMSE score and education (n=2510; mean± a 95% confidence interval): red, women; dark green, men. The Pearson coefficient=0.9115 in men; =0.8412, in women.

Given the above associations between education and healthy cognition, an adjustment for education was applied to assess the impact of education on the GWAS results (Table S2, Figure S3).

**Table S2.** GWAS results, MMSE as a continuous variable, adjusted for education

| Chr | Position | AF | LR coefficient | p-value | Statistics in the Hardy-Weinberg test | Hardy-Weinberg test  (p-value) | Gene | Gene variant | snp |
| --- | --- | --- | --- | --- | --- | --- | --- | --- | --- |
| chr19 | 44908684 | 0.077 | -2.41 | 3.96*10^-12^ | 2.88 | 0.24 | APOE | missense variant | rs429358 |
| chr19 | 27353865 | 0.014 | -4.29 | 3.13*10^-8^ | 0.55 | 0.76 |  | intergenic variant | rs145461979 |
| chr19 | 44912456 | 0.063 | -2.42 | 1.89*10^-10^ | 1.03 | 0.60 | APOE | downstream gene variant | rs10414043 |
| chr19 | 44906745 | 0.061 | -2.42 | 3.02*10^-10^ | 1.51 | 0.47 | APOE | intron variant | rs769449 |
| chr19 | 27355589 | 0.026 | -3.21 | 3.59*10^-8^ | 1.88 | 0.39 |  | intergenic variant | rs113472381 |
| chr19 | 27369272 | 0.018 | -4.35 | 7.68*10^-10^ | 0.82 | 0.66 |  | intergenic variant | rs10048455 |
| chr10 | 106063954 | 0.012 | -5.09 | 3.51*10^-9^ | 0.35 | 0.84 |  | intergenic variant | rs193174984 |

Chr: chromosome number; AF: allele frequency; LR coefficient: logistic regression coefficient


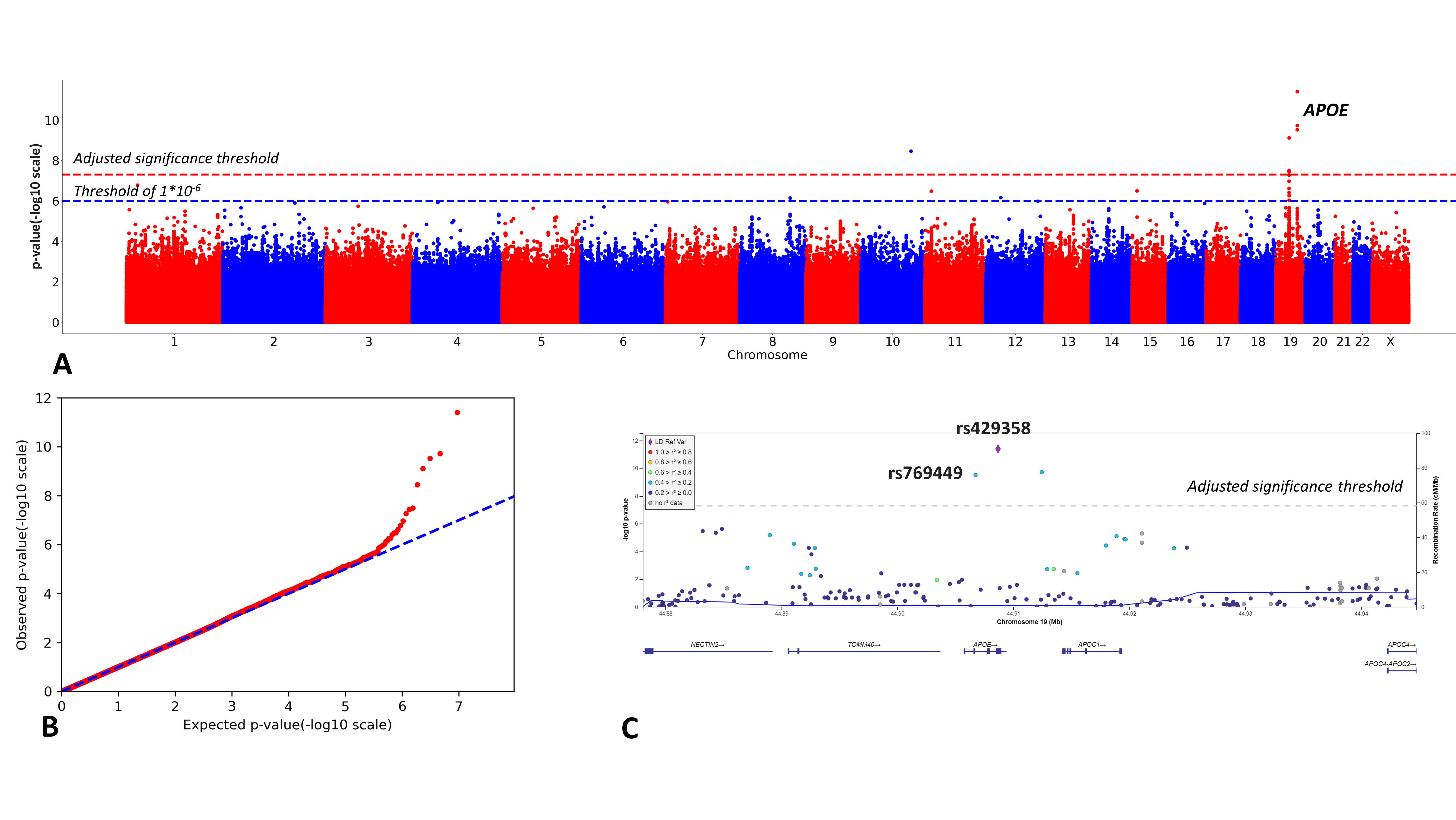


***Figure*** S3. Manhattan plot (A), QQ plot (B) and regional association plot (C) for the linear regression model of the MMSE scores adjusted for education.

*A: Manhattan plot of the –log10 p-values of the common variants (major allele frequencies of >0.01). The dashed red line represents a Bonferroni threshold of (-log10(5*10-8)). The dashed blue line represents a threshold of (-log10(1*10-6)).*

*B: QQ-plot for performed GWAS. Most P-values were similar to the expected diagonal in the QQ-plot, which indicates the appropriateness of the GWAS model.*

*C - Regional association plot for the locus on chromosome 19 (chromosome 19:44878048-44944779) that contains all significant results. The most strongly associated SNP (rs429358, the lead) located in the gene APOE is represented by the purple dot. The color indicates the strength of linkage disequilibrium between the lead SNP rs429358 and other SNPs in this region. Dashed line represents a Bonferroni threshold of (-log10(5*10-8)). The data were visualized with LocusZoom*

**APOE protein modelling**


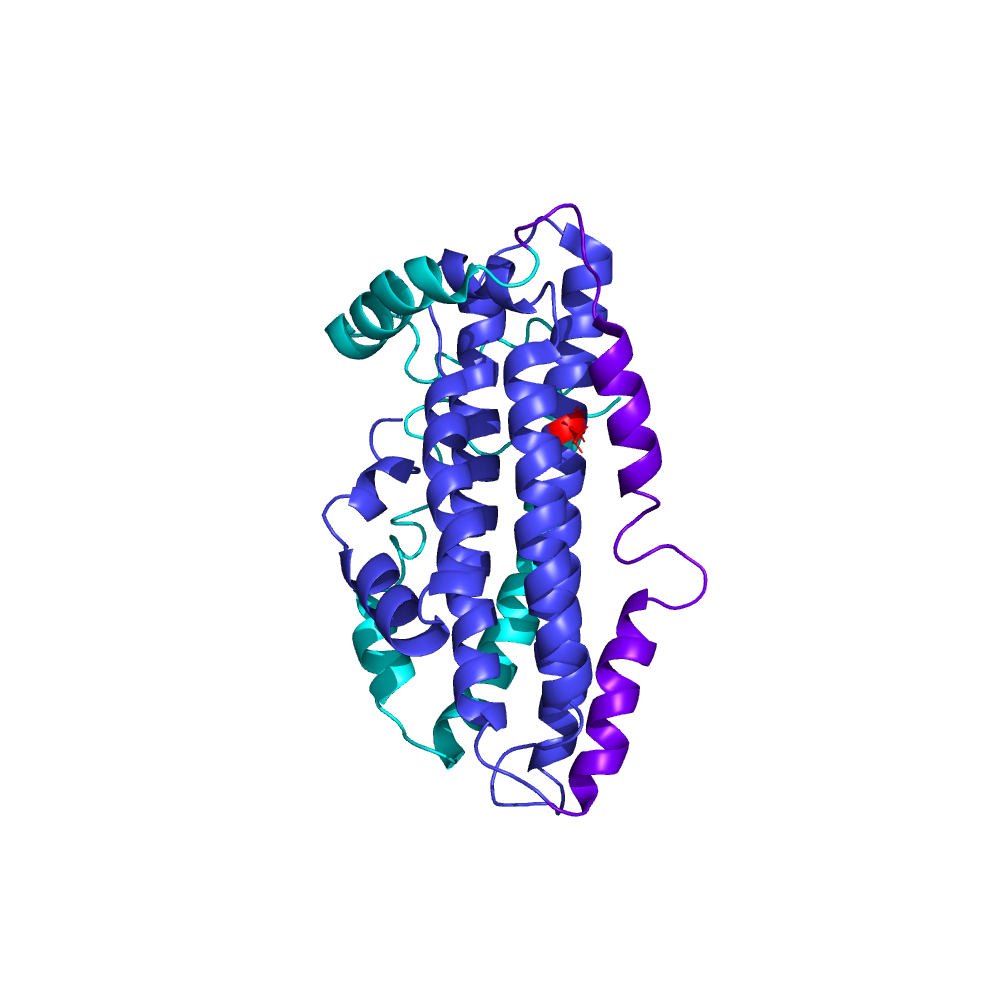


***Figure S4.*** *The PDB:2L7B structure (APOE ε3 allele). The rs429358 substitution site at aminoacid 112 is shown in red. The color backgrounds demarcate the domains: purple, the N-terminal domain; beige, the hinge domain; cyan, the С-terminal domain.*

**APOE genotype analysis**

**Table S3.** Associations between APOE genotypes, lipid metabolism, and one-year mortality.

| Factor | ε2 carriers | | ε4 carriers | |
| --- | --- | --- | --- | --- |
|  | coef | p-value | coef | p-value |
| Cholesterol* | -0.36 | 2.41*10^-9^ | 0.169 | 0.013 |
| LDL* | -0.429 | 1.38*10^-17^ | 0.188 | 9.2*10^-4^ |
| HDL* | 0.04 | 0.026 | -0.006 | 0.76 |
| LDL/HDL* | -0.413 | 2.47*10^-17^ | 0.194 | 4.2*10^-4^ |
| Triglycerides* | 0.0424 | 0.157 | -0.0222 | 0.509 |
| One-year mortality** | -0.295 | 0.054 | 0.575 | 2.4*10^-10^ |

*All computation results were adjusted for age and sex: *linear regression (n=2,525), ** logistic regression (n=1,350).*

**Table S4.** Distribution of APOE alleles and effects of APOE genotypes on cognitive status

| ***APOE genotype*** | **Entire cohort**  **n=2559** | **Cognitive impairment**  **(MMSE<10)**  **n=179** | **No cognitive impairment**  **(MMSE>24)**  **n=976** | **OR (adjusted for age and sex)** | **p-value (adjusted for age and sex)** |
| --- | --- | --- | --- | --- | --- |
| ε2/ε2 | 22 (1%) | 0 (0%) | 7 (0.7%) | (N/A) | (N/A) |
| ε2/ε3 | 436 (17%) | 23 (13%) | 193 (19.8%) | 0.57 | 0.018 |
| ε3/ε3 | 1718 (67%) | 109 (61%) | 671 (68.8%) | 0.7 | 0.035 |
| ε3/ε4 | 332 (13%) | 40 (22.3%) | 92 (9.4%) | 3.15 | 1.16*10^-7^ |
| ε2/ε4 | 42 (1.6%) | 6 (3.4%) | 12 (1.2%) | 2.65 | 0.056 |
| ε4/ε4 | 9 (0.4%) | 1 (0.6%) | 1 (0.1%) | (invalid) | (invalid) |


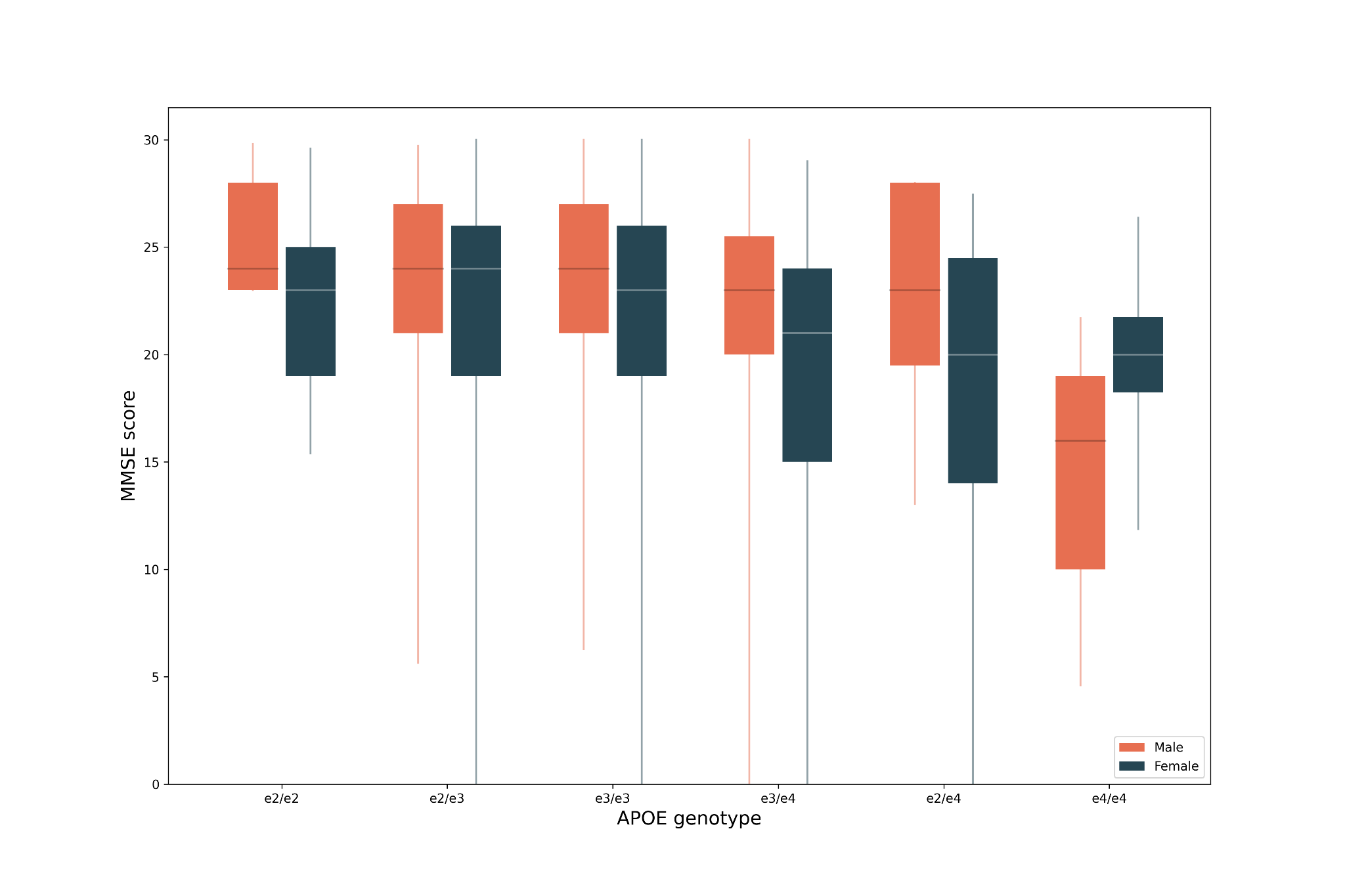


**Figure S5.** Effects of APOE genotypes on MMSE scores in men and women.

**Polygenic risk score model**

**Table S5**. Polygenic risk score

| **Factor** | **Coefficient of logistic regression** | **Annotation** |
| --- | --- | --- |
| age | 0,002214317665982 |  |
| gender | 0,245215096490867 | 0 - male, 1 - female |
| rs199636355 | 0,275422229877398 | intergenic |
| rs113859335 | 0,195016448961114 | GRIK3 : Intron Variant |
| rs10172559 | 0,078836822982092 | intergenic |
| rs1293508533 | -0,058696365340298 | intergenic |
| rs1744644195 | 0,189745876961246 | SV2C : Intron Variant |
| rs114340769 | 0,155875876111888 | intergenic |
| rs149526603 | 0,015689537546634 | intergenic |
| rs147216969 | 0,062325404693582 | intergenic |
| rs76321024 | 0,126323724512732 | intergenic |
| rs558789972 | 0,252353226051188 | LOC101927413 : Intron Variant |
| rs74648183 | 0,230672037319957 | DKK3 : Intron Variant |
| rs77051199 | 0,282076291358938 | intergenic |
| rs142796611 | 0,156250624197996 | intergenic |
| rs113904098 | 0,024887892134426 | ATP8B1 : Intron Variant |
| rs62108629 | -0,03643653139445 | intergenic |
| rs113288717 | 0,12542168575086 | intergenic |
| rs145461979 | -0,036545132416901 | intergenic |
| rs113472381 | 0,147490667524558 | intergenic |
| rs74358270 | 0,032397859659572 | intergenic |
| rs76931914 | 0,014626007056575 | intergenic |
| rs73517494 | 0,078297454686737 | intergenic |
| rs4556913 | 0,036183527122366 | intergenic |
| rs8190046 | 0,076330280151749 | intergenic |
| rs61142671 | 0,089357615962671 | intergenic |
| rs78563345 | 0,146534464532009 | intergenic |
| rs3990609 | 0,180215404390351 | intergenic |
| rs10048455 | 0,01170225600688 | intergenic |
| rs74860125 | 0,067201654992055 | intergenic |
| rs79211561 | -0,017642104843833 | intergenic |
| rs2407329 | 0,042542255757877 | intergenic |
| rs879684591 | 0,144006539016068 | intergenic |
| rs903534648 | 0,303744396765785 | LOC124904698 : 500B Downstream Variant |
| rs1555789087 | 0,107950162936296 | TOMM40 : Intron Variant |
| rs769449 | 0,072070702470871 | APOE : Intron Variant |
| rs429358 | 0,158267302681639 | APOE : Missense Variant |
| rs10414043 | 0,086207074361425 | APOC1 : 2KB Upstream Variant |
| rs7256200 | 0,086207074361425 | APOC1 : 2KB Upstream Variant |
| rs148698960 | 0,053375565420977 | intergenic |
| rs57424378 | 0,049303248283308 | intergenic |
| rs2147478407 | 0,049303248283309 | intergenic |
| rs73942479 | 0,049303248283308 | intergenic |
| rs12459697 | 0,049303248283308 | intergenic |
| rs73942481 | 0,070011962380957 | intergenic |
| rs1489427270 | 0,137789405829771 | intergenic |
| rs2945205 | 0,16300765295643 | intergenic |

**Pathway enrichment analysis**

The intGSASNP R-package was used for functional analysis of gene ontology biological process (GO BP) enrichment (1). Stouffer method with LD correction was applied to sets of single-nucleotide polymorphisms (SNPs) within each gene to generate a combined p-value at the gene-level (1). The Coincident Extreme Ranks in Numerical Observations (CERNO) algorithm was used for gene set analysis (2).


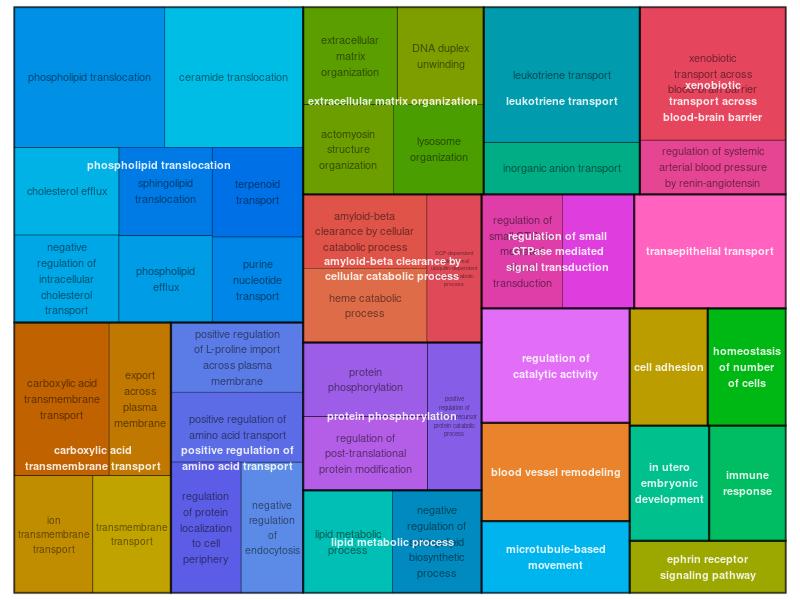


Figure S6. Results of the pathway enrichment analysis

Most polymorphism-enriched pathways are involved in the xenobiotic transfer across the cell membrane and transport of lipids. Changes in ATP-binding cassette (ABC) transporters, which are involved in the transport of compounds across the blood-brain barrier, may be associated with changes in animal behavior (3), aging (4), and the development of neurodegenerative diseases, resulting, among others, from an APOE-mediated mechanism (5).

Results of functional analysis can only indicate which pathway is significantly enriched with polymorphisms, but cannot determine how the pathway changes. Thus, we can conclude that CI in the long-living adults in Moscow and the Moscow region may be associated with the pathway of xenobiotic transport through the blood-brain barrier, but we cannot specifically state whether the transport accelerated or decelerated. This topic needs further research.

**References:**

1. Marczyk M, Macioszek A, Tobiasz J, Polanska J, Zyla J. Importance of SNP Dependency Correction and Association Integration for Gene Set Analysis in Genome-Wide Association Studies. Front Genet. 2021;12: 767358.
2. Zyla J, Marczyk M, Domaszewska T, Kaufmann SHE, Polanska J, Weiner J. Gene set enrichment for reproducible science: comparison of CERNO and eight other algorithms. Bioinformatics. 2019;35. doi:10.1093/bioinformatics/btz447.
3. Hindle SJ, Munji RN, Dolghih E, Gaskins G, Orng S, Ishimoto H, et al. Evolutionarily Conserved Roles for Blood-Brain Barrier Xenobiotic Transporters in Endogenous Steroid Partitioning and Behavior. Cell Rep. 2017;21: 1304–1316.
4. Efferth T. Adenosine triphosphate-binding cassette transporter genes in ageing and age-related diseases. Ageing Res Rev. 2003;2. doi:10.1016/s1568-1637(02)00046-6
5. Rawat V, Wang S, Sima J, Bar R, Liraz O, Gundimeda U, et al. ApoE4 Alters ABCA1 Membrane Trafficking in Astrocytes. J Neurosci. 2019;39: 9611–9622.
